# Staphylococcal Enterotoxin C Is an Important Virulence Factor for Mastitis

**DOI:** 10.3390/toxins11030141

**Published:** 2019-03-02

**Authors:** Rendong Fang, Jingchun Cui, Tengteng Cui, Haiyong Guo, Hisaya K. Ono, Chun-Ho Park, Masashi Okamura, Akio Nakane, Dong-Liang Hu

**Affiliations:** 1 College of Animal Science and Technology, Southwest University, Congqing 400715, China; rdfang@swu.edu.cn; 2 College of Life Science, Dalian Minzu University, Dalian 116600, China; tengteng.cui@pharmaron-bj.com (T.C.); guohaiyong78@jlnu.edu.cn (H.G.); 3 Department of Biological Science, School of Life Science, Jilin Normal University, Siping 136000, China; 4 Department of Microbiology and Immunology, Hirosaki University Graduate School of Medicine, Hirosaki, Aomori 036-8562, Japan; hisaono@vmas.kitasato-u.ac.jp (H.K.O.); a27k03n0@hirosaki-u.ac.jp (A.N.); 5 Kitasato University School of Veterinary Medicine, Towada, Aomori 034-8628, Japan; baku@vmas.kitasato-u.ac.jp (C.-H.P.); okamuram@vmas.kitasato-u.ac.jp (M.O.)

**Keywords:** staphylococcal enterotoxin, mastitis, superantigen, virulence factor

## Abstract

*Staphylococcus aureus* is an important bacterial pathogen causing bovine mastitis, but little is known about the virulence factor and the inflammatory responses in the mammary infection. Staphylococcal enterotoxin C (SEC) is the most frequent toxin produced by *S. aureus*, isolated from bovine mastitis. To investigate the pathogenic activity of SEC in the inflammation of the mammary gland and the immune responses in an animal model, mouse mammary glands were injected with SEC, and the clinical signs, inflammatory cell infiltration, and proinflammatory cytokine production in the mammary glands were assessed. SEC induced significant inflammatory reactions in the mammary gland, in a dose-dependent manner. SEC-injected mammary glands showed a severe inflammation with inflammatory cell infiltration and tissue damage. In addition, interleukin (IL)-1β and IL-6 production in the SEC-injected mammary glands were significantly higher than those in the PBS control glands. Furthermore, the SEC-induced inflammation and tissue damage in the mammary gland were specifically inhibited by anti-SEC antibody. These results indicated, for the first time, that SEC can directly cause inflammation, proinflammatory cytokine production, and tissue damage in mammary glands, suggesting that SEC might play an important role in the development of mastitis associated with *S. aureus* infection. This finding offers an opportunity to develop novel treatment strategies for reduction of mammary tissue damage in mastitis.

## 1. Introduction

Bovine mastitis is one of the most prevalent diseases which represents a huge economic loss for the dairy industry and is an increasing public health concern, worldwide [1,2,3]. *Staphylococcus aureus* is a common bacterial pathogen, isolated frequently from the milk produced by cows with mastitis, and is also an important bacterium causing bovine mastitis [4,5]. *S. aureus* can enter the mammary gland, adhere to epithelial cells, and start growing there, resulting in a mammary epithelial line damage that is initiated by the infiltration of inflammatory cells in the parenchyma, the alveoli, causing ulceration and occlusion of lactiferous ducts [6]. To treat acute inflammation of the udder tissue and improve animal health, cows have to be administrated with antibiotics, and the milk production becomes impaired for a long period and results in a huge economic loss. It is essential to reduce the prevalence of bovine mastitis infected with *S. aureus*, by different measures, such as vaccination and immunotherapy [4]. The clinical outcome of acute mastitis and progression to persistent mastitis might be related to the presence of virulence factors of *S. aureus* strains causing the infection. During the past decade, many studies have attempted to develop vaccines against some specific virulence factors that were considered to be responsible for the development and persistent infection of *S. aureus*-induced bovine mastitis. However, the vaccines have shown a limited protective efficacy in the dairy farm sites [7,8,9]. The lack of effective vaccines could be attributed, in part, to the incomplete understanding of the pathogenic mechanism of mastitis induced by *S. aureus* and the mechanisms of immune responses in the ruminant breasts [10].

Many *S. aureus* isolated from bovine mastitis harbor genes coding for superantigenic toxins, such as staphylococcal enterotoxins (SEs). SEs are members of the pyrogenic toxin family, including classic toxins, staphylococcal enterotoxin A (SEA) to SEE, and newly described toxins, SEG to SEI, SEK to SET, and staphylococcal enterotoxin like toxin J (SElJ), and SElU to SElY. The superantigenic toxins show strong T cell mitogenic activity by directly binding to major histocompatibility complex (MHC) class II molecules of antigen presenting cells and Vβ regions of T cell receptor (TCR), without the normal antigen presentation process [11,12,13]. Large amounts of activated cells release excessive inflammatory cytokines, such as interleukin-2 (IL-2), tumor necrosis factor alpha (TNF-α), and gamma interferon (IFN-γ), which are responsible for the development of inflammation, rashes, fever, multiorgan damage, and toxic shock syndrome, in humans and animals [14,15,16]. Previous studies have reported that *S. aureus* isolated from bovine mastitis produce superantigenic toxins, especially SEC [17,18]. SEC has also been detected in the *S. aureus* strains isolated from an outbreak case of skin and soft tissue infections, including mastitis in humans [19]. Several studies reported that the *sec* gene was frequently detected in *S. aureus* strains isolated from raw milk of goats and bovines with mastitis [20,21,22]. Although epidemiological studies showed that the presence of *sec* gene in the majority of strains of bovine mastitis and suggested a possible involvement of SEC in bovine mastitis pathogenesis [4,23,24,25], It is still unknown whether or not SEC is a direct virulence factor for bovine mastitis [25,26,27].

To understand the role of SEC in the development of bovine mastitis, we cloned and expressed the *sec* gene in the prokaryotic expression system, and the biological activities of purified SEC were analyzed in the present study. The pathogenicity of SEC and the immune responses of mammary gland induced by SEC were studied using the mouse model. Our results demonstrated that SEC can induce inflammatory reaction, cytokine production, and tissue damage in the mammary gland, indicating that SEC could be an important virulence factor for mastitis.

## 2. Results

### 2.1. Biological Activities of Purified Recombinant SEC

**Figure 1:**
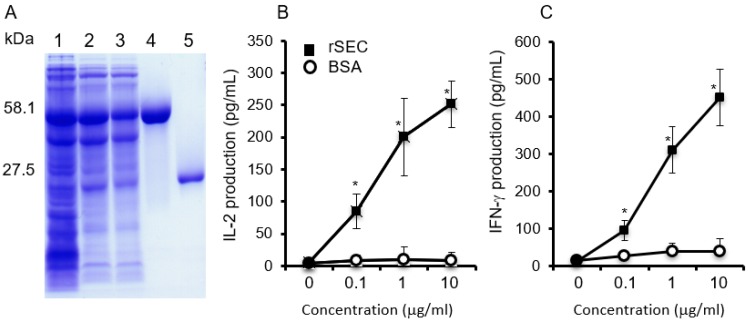
Biologic and superantigenic activities of purified staphylococcal enterotoxin C (SEC). (**A**) Purified SEC analyzed by SDS-PAGE. Lanes 1–3, supernatants of *E. coli* encoding glutathione S-transferase (GST)-SEC; lane 4, purified GST-SEC fusion protein; lane 5, purified SEC. (**B**,**C**) Production of cytokines in mouse spleen cell cultures stimulated with 0.1, 1, and 10 μg per mL of SEC (3.6, 36, and 360 nM) or BSA (1.5, 15, and 150 nM). Amounts of IL-2 (**B**) and IFN-γ (**C**) in the spleen cell cultures were measured by ELISA. Results are the mean ± SD, based on the samples obtained from five to six mice. Two experiments were performed independently. An asterisk indicates that the value was significantly different between the SEC and the BSA control group at *p* < 0.05.

The biological and superantigenic activities of purified SEC were identified and assayed. Analysis of SEC by Coomassie-blue-stained SDS-PAGE revealed the presence of a purified protein band that was readily detectable (Figure 1). The level of endotoxin in the final purified SEC was not detected (less than 0.001 EU/mL). The superantigenic activity of SEC was evaluated by sandwich ELISA to determine the production of IL-2 and IFN-γ in mouse spleen cell culture supernatants. Compared with the BSA control group, the SEC-induced spleen cells produced large amounts of IL-2 and IFN-γ, in a dose dependent manner (Figure 1).

### 2.2. SEC Exhibited Pathogenic Activity in the Murine Mammary Glands

**Figure 2:**
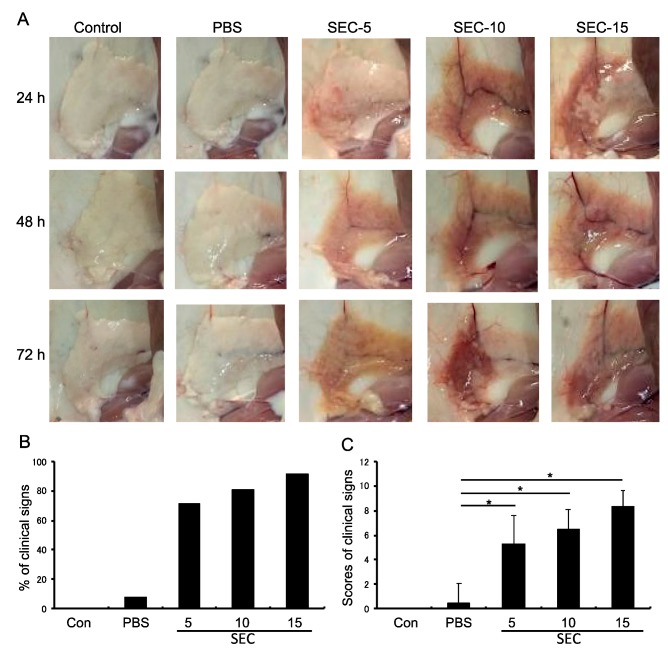
Appearance observation of mouse mammary glands injected with PBS, SEC (5, 10 or 15 μg per gland) or non-treated controls. (**A**) Mice were euthanized 24, 48, and 72 h after injection, and investigated for the extent of inflammation of the mammary gland area by observing the clinical signs, including redness, swelling, congestion, bleeding, and discoloration of the mammary glands. The results are representative of three independent experiments. (**B**) Percentages of mice with clinical symptoms for each group. (**C**) Scores of clinical signs of mice for each group. An asterisk indicates that the value was significantly different between the SEC and the PBS control group at *p* < 0.05.

To study whether SEC could induce mastitis in the murine mammary glands, mice were injected with PBS, SEC (5, 10, or 15 μg per gland), and non-treated normal control (Figure 2). Results showed that SEC significantly induced inflammatory responses and tissue damage in the mammary glands, 24 h after injection with the toxin (Figure 2). The pathogenic activity of SEC was found to be dose-dependent. The percentages of mice with clinical signs, including redness, swelling, congestion, bleeding, and discoloration of the mammary glands, were recorded and calculated. The mice injected with SEC at 5, 10, and 15 μg per gland showed 71.4% (10/14), 80.0% (12/16), and 91.7% (11/12) positive clinical signs, respectively. In contrast, the mice injected with PBS showed 7.7% (1/13) positive clinical signs (Figure 2B). The scores of clinical signs of mice for each group are shown in Figure 2C. The inflammation signs were still observed 72 h after SEC injection. In contrast, the PBS-treated and non-treated control mice showed no significant changes in the mammary glands.

### 2.3. Histopathological Changes of SEC-Injected Mammary Glands

**Figure 3:**
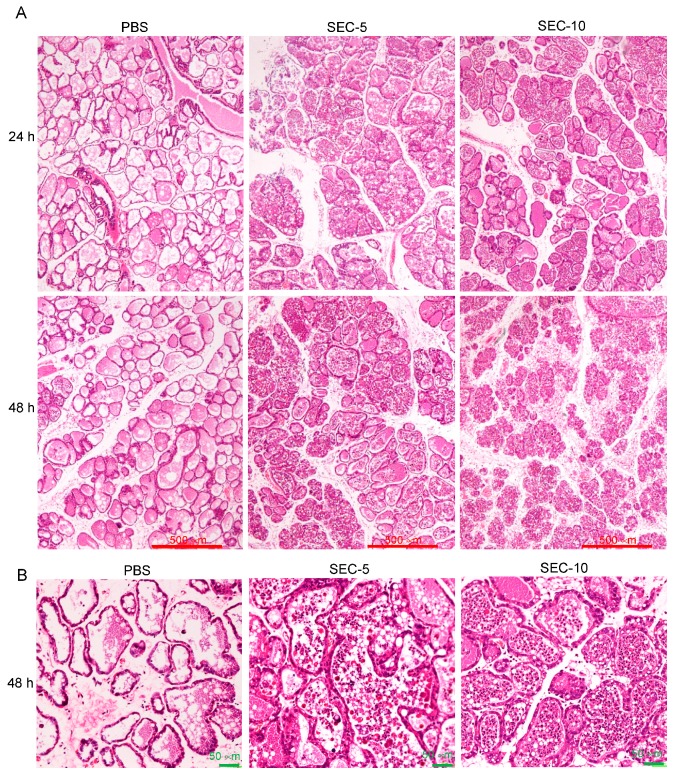
Histological changes of mammary glands of mice injected with PBS or SEC (5 or 10 μg per gland). The mammary glands were collected and the right glands were fixed in 10% neutral buffered formalin for 24 h, processed with automated tissue processor and embedded in paraffin wax. Sections were cut at three levels to a thickness of 4 μm and stained by HE staining. The results are representative of three independent experiments. (**A**) Low magnification photographs and the bars show 500 μm. (**B**) High magnification photographs and the bars show 50 μm.

Histopathological changes in the mammary glands injected with SEC or PBS were evaluated by hematoxylin and eosin (HE) staining (Figure 3). No significant abnormality was observed in the histopathological observation of the breast tissue of the non-treated group (data not shown). The PBS treated group with little histopathological change was almost identical to the untreated control group. In the SEC-treated group, there were inflammatory cells including neutrophils, macrophages, and monocytes infiltrating the mammary gland tissues. Quantification of the number of inflammatory cells, including neutrophils, macrophages, and monocytes, showed that the numbers of cells in mammary glands injected with PBS, 5 or 10 µg of SEC per gland, were 7 ± 4, 71 ± 12, and 80 ± 16 per field of view, respectively. The number of inflammatory cells in the mammary glands after injection with 5 and 10 µg SEC were significantly higher than that of the PBS control group (*p* < 0.05). In addition, interstitial edema, thickening of the gland alveolus wall and acutely damaged alveolar form were also observed (Figure 3). 

### 2.4. SEC Induced the Production of Proinflammatory Cytokines

**Figure 4:**
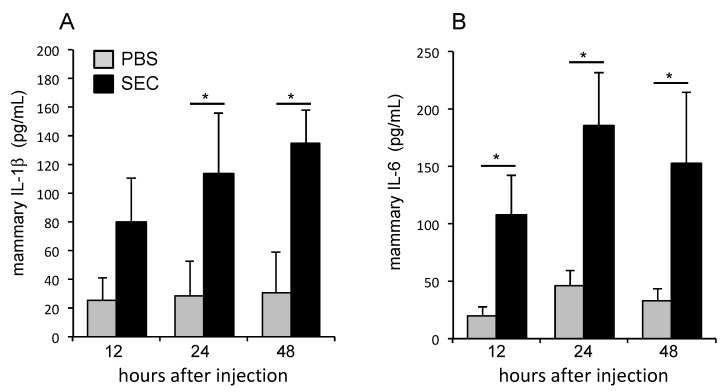
Production of proinflammatory cytokine in mammary glands of mice injected with PBS or SEC (10 μg per gland). Mammary tissues were taken out from each group, weighed and homogenized with PBS. Homogenate was removed into a centrifuge tube and centrifuged at 3,000 rpm, for 20 min, at 4 °C. The amounts of IL-1β (**A**) and IL-6 (**B**) in the supernatant of the homogenate were analyzed with the corresponding ELISA. The results are representative of three experiments, and the data are the mean ± SD for groups of three to five mice. An asterisk indicates that the value was significantly different between SEC-injected group and PBS control group at *p* < 0.05.

To evaluate the inflammatory and immune responses of the mammary glands injected with SEC, proinflammatory cytokines, IL-1β and IL-6, were measured in SEC-treated (10 μg/gland) or PBS-treated glands at 12, 24, and 48 h, after mammary injection (Figure 4). SEC-injection caused a significant increase of IL-1β and IL-6 in the mammary tissue of mice, compared with that of the control group at 12, 24, and 48 h, after mammary injection. Higher levels of cytokines were recorded in the SEC-treated glands which also showed neutrophils, macrophages, and monocytes infiltration during the histological observation. The results were consistent with the histological data and indicated the recruitment of immune cells in the alveolar lumen, at an earlier time point, in the mammary glands exposed to SEC.

### 2.5. Anti-SEC Antibody Inhibits the Inflammation of Mammary Gland Induced by SEC

**Figure 5:**
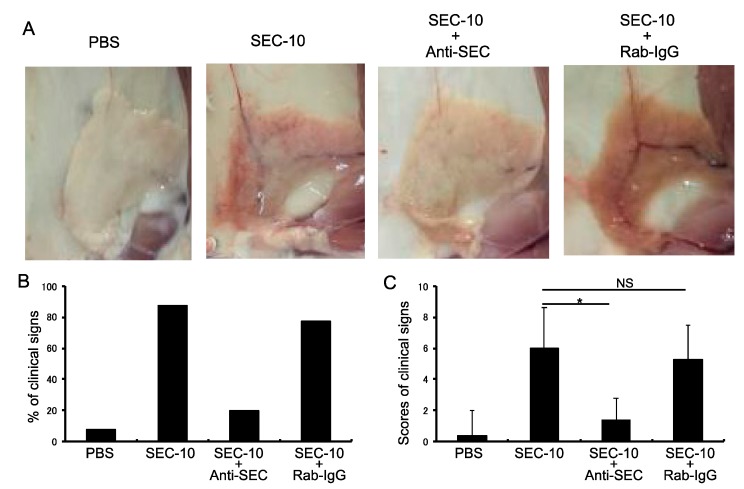
Neutralization ability of anti-SEC antibody against the inflammation of mammary gland induced by SEC in vivo. (**A**) Rabbit anti-SEC IgG or normal rabbit IgG, preincubated with SEC at 37 °C, for 1 h before SEC (10 μg per gland) were injected into the mammary glands of mice. The injected mice were then investigated for the extent of inflammation of the mammary gland at 48 h after injection, by observing clinical signs, swelling, redness, congestion, and bleeding of the mammary glands. The results are representative of two independent experiments, each with three to five mice per each group. (**B**) Percentages of mice with clinical symptoms for each group. (**C**) Results showing the clinical signs in mice for each group. An asterisk indicates that the value was significantly different between the SEC + anti-SEC IgG group and the SEC alone control group at *p* < 0.05; NS indicates no significant difference.

To further investigate whether the SEC-induced inflammation in mammary gland could be inhibited by anti-SEC antibody, SEC was preincubated with rabbit anti-SEC IgG, or with normal rabbit IgG, and then injected to the mammary glands of mice (Figure 5). The numbers and percentages of mice with clinical signs, including redness, swelling, congestion, bleeding, and discoloration of the mammary glands, were recorded and analyzed. The mice injected with SEC alone, SEC + anti-SEC IgG, or SEC + normal rabbit IgG showed an 87.5% (7/8), 20.0% (2/10), and 77.7% (7/9) positive clinical signs, respectively (Figure 5B). The results of the clinical signs for the mice of each group are shown in Figure 5C. The inflammatory reactions of the mammary glands were markedly inhibited by treatment with the anti-SEC antibody, compared with the SEC alone and SEC + normal rabbit IgG groups (Figure 5).

## 3. Discussion

*S. aureus* strains isolated from the bovine mastitis cases carried multiple genes encoding SEs or SEls, especially SEC, has been reported extensively. Previous studies have also reported that *S. aureus* pathogenicity island (SaPI), containing *sec* and *sel*, was detected in isolates of mastitis outbreak in cows, goats, and humans, indicating that *sec* and *sel* carrying *S. aureus* might be important and might be associated with mastitis in animals and humans [4,28,29,30]. However, little is known regarding if SEC is a virulence factor for bovine mastitis induced by *S. aureus*. In the present study, we analyzed the biological characteristics and potential pathogenic activity of SEC in mouse mastitis model that were directly injected with a purified SEC. Our results revealed that SEC, a superantigenic toxin, induced markedly inflammatory reactions and tissue damage in the mammary glands of the SEC-injected mice. These results indicated, for the first time, that SEC is an important virulence factor which contributes for occurrence and deterioration of mastitis.

Analysis of histopathological changes of mammary tissue is a widely used method to evaluate mammary tissue damage caused by bacterial invasion [6,31]. In the present study, SEC-induced acute mastitis was assessed, based on macroscopic clinical manifestations, histopathological changes, inflammatory cytokine production, and the level of mammary tissue damage. The inflammation with tissue degeneration and necrosis, and inflammatory cell infiltration in the intra-mammary tissue induced by SEC were observed. Moreover, SEC-induced inflammation and tissue damage were significantly inhibited by treatment of SEC with anti-SEC antibody, before SEC injection. Previous studies have demonstrated that *S. aureus*-induced bovine mastitis showed characteristic histopathological changes, including inflammatory cell infiltration, shrinkage of alveolar space, damage of secretory breast tissue, and necrosis of the mammary gland [32,33]. Inflammatory cells, such as neutrophil, macrophage, and monocyte play an important role in the host defenses against *S. aureus* invasion of the mammary gland [34]. On the other hand, however, excessive infiltration of inflammatory cells can also damage the mammary epithelia lines by respiratory burst and degranulation of the cells [35]. SEC-induced inflammatory reactions in mammary glands could be due to a large amount of inflammatory cell infiltration, excessive respiratory bursts, and degranulation and destruction of the mammary epithelial cells.

Inflammatory cytokines are vital mediators of the development and persistence of inflammation during mammary infections. Previous studies have reported that a large amount of IL-1 and TNF-α in milk and plasma of animals with mastitis might be important events for the inflammatory reactions in mammary glands [36,37,38]. The high level of IL-1 and TNF-α in the serum, could induce the activation and migration of neutrophils, and cause apoptosis of endothelial cells and mammary epithelial cells, in bovine and human suffering from acute clinical mastitis [39,40,41,42]. Inflammatory cytokines also increase a wide variety of functions of the inflammatory cells, including cell adhesion, expression of cell surface receptors, release of lysosomal constituents, and free radical production [34]. In the present study, the levels of proinflammatory cytokines, IL-1β and IL-6, were significantly higher in the mammary glands of mice injected with SEC than those of the control mice injected with PBS, indicating that the cytokines play an important role in the observed mammary tissue damage in mice. Although superantigen might generate varying effects, depending on the animal species [43,44], the effect of SEC-induced cytokines on breast tissue injury is likely to be mediated by the recruitment and activation of inflammatory cells in the mammary gland.

In conclusion, our results showed that mammary gland injected with SEC markedly caused inflammatory reactions, production of inflammatory cytokines, and tissue damage, in the mammary gland. To our knowledge, the present study is the first demonstration that SEC could directly induce mastitis in an animal model. Together with the molecular epidemiological data showing that the sec gene is frequently detected in isolates of bovine mastitis, our results suggest that SEC contributes to the pathogenesis through a superantigenic activity, which induces proinflammatory cytokine release, inflammation responses, and subsequently induces mammary tissue damage. This finding provides an opportunity to develop novel treatment strategies to alleviate tissue damage in the mammary glands infected by bacteria.

## 4. Materials and Methods 

### 4.1. Expression and Purification of SEC

The *sec* gene was amplified from the genomic DNA of *S. aureus* 834 strain, which is a clinical isolate that produces SEC and induces mastitis, in the mouse model [45,46]. The PCR primers used were SEC/GST+ (5′-CCCCGGATTCGAGAGCCAACCAGACCCTACG), including a *BamH*I site, and SEC/GST- (5′-CCCCGAATTCTTATCCATTCTTTGTTGTAAGGTGG), including an *EcoR*I site, and the predicted size of the PCR product was 669 bp. The PCR products were subcloned into a pGEX-6P-1 glutathione S-transferase (GST) fusion vector. The expression and purification of the recombinant SEC were performed, as described by Hu et al. [47]. Purified SEC protein was analyzed by sodium dodecyl sulfate polyacrylamide gel eletrophoresis (SDS-PAGE) and the Bradford method (Bio-Rad Laboratories, Richmond, CA, USA). Endotoxin contamination in the purified proteins was removed by ProteoSpin™ Total Protein Concentration, Detergent Clean-Up, and Endotoxin Removal Kit (Norgen Biotek Corp., Thorond, ON, Canada), and was detected by Limulus amebocyte lysate PYROTELL (CAPE COD, Falmouth, MA, USA).

### 4.2. Assay of Superantigenic Activity of SEC

To analyze the superantigenic activity of purified SEC, five six-week-old Balb/c mice were used for the spleen cell culture experiment. Analysis of the production of IL-2 and IFN-γ in the spleen cell cultures induced by the toxin were performed, as described previously [48]. The amount of IL-2 and IFN-γ in the supernatant of the cell cultures were determined by sandwich ELISAs, as described by our previous reports [47,48].

### 4.3. Intramammary Inoculation Model

Ten-week-old healthy Kunming mice were purchased from the Experimental Animal Center of Dalian Medical University. The mice were fed with a special diet for breeding, and drank freely. After one week of adaptive feeding, two female and one male mice were fed in the same cage to allow them to mate. The pregnant female mice were randomly divided into seven groups of 5 mice each, and used for the injection experiment. Injection of SEC into mammary glands was carried out using a modification to the procedure described previously [31,44]. Mice were anesthetized with ketamine (100 mg/kg) and xylazine (10 mg/kg) by intraperitoneal injection. With the mouse facing up, the mammary gland and its periphery were sterilized with 70% ethanol. A total of 20 μL of SEC was injected directly into the mammary duct, using a 31-gauge hypodermic needle to a depth of not more than 4 mm, without cutting or scraping the shallow layer of the mammary gland. The mice were observed to assess development of clinical signs of mastitis. For a control group, mice were injected with 20 μL of PBS, using the same procedure. The animal experiments described in this study were approved by the animal ethics committee of Daliang Minzu University (27 July 2015; DMU2015-002) and carried out in accordance with the guide for the management and use of experimental animals of Dalian Medical University.

### 4.4. Clinical Evaluation

The changes in mammary glands of mice injected with SEC were observed and evaluated for onset of mastitis. Clinical signs, swelling, redness, and discoloration of the breast were observed and recorded. Monitoring of mice for morbidity and mortality was carried out up to 72 h. Mice were euthanized at 24, 48, and 72 h after injection and investigated for the extent of inflammation of the mammary gland area, by observing redness, swelling, congestion, bleeding, and discoloration of the mammary gland. A total score of 10 could be obtained for the 5 symptoms, with 2 for each positive clinical sign and 0 for each negative clinical sign.

### 4.5. Histopathological Examination 

To estimate the inflammation levels and histological changes, the mammary gland tissue of each group was collected and the right gland was fixed in 10% neutral buffered formalin, for 24 h, before being processed with an automatic tissue processor, and was embedded in paraffin. Sections were cut at three levels to a thickness of 4 μm and stained by the HE staining. The number of inflammatory cells, including neutrophils, macrophages, and monocytes, in the field of view were counted and quantified. Five fields of view of each sample were screened to obtain an average number of an animal, then the total mean and standard deviation of each group were calculated and analyzed.

### 4.6. Determination of Cytokines 

To determinate cytokine production in the mammary gland, mammary tissues of each experiment group were taken out, weighed, and homogenized with PBS with 1:9 ratio. After the tissue was grinded, radically, the homogenate was centrifuged at 3,000 rpm, for 20 min, at 4 °C. The supernatant was stored at −30 °C. The contents of important inflammatory cytokines in the mammary gland, IL-1β and IL-6, were analyzed by the corresponding ELISA kits, according to the instructions of the manufacturers (BioLegend, Inc., Camino Santa Fe, San Diego, CA, USA). 

### 4.7. Neutralization Assay of the Anti-SEC Antibody

To analyze the neutralizing activity of anti-SEC antibody, against the inflammation of mammary gland, induced by SEC, in vivo, 300 μL of rabbit anti-SEC IgG (1 mg/mL) or normal rabbit IgG (1 mg/mL), as a control antibody, were preincubated with 300 μL of SEC (1 mg/mL) at 37 °C, for 1 h, before the SEC was injected to the mammary gland. Twenty microliters of the preincubated solution, including 10 μg of antibody and 10 μg of SEC, was injected directly into the mammary duct, using a 31-gauge hypodermic needle. Both antibodies were prepared previously in our laboratory [45,46]. Mice were investigated for the extent of inflammation of the mammary gland area, 48 h after injection, by observing the clinical signs, swelling, redness, congestion, and bleeding of the mammary gland, as described above.

### 4.8. Statistical Analysis 

Data in this study were showed as means ± standard deviations. The significance of differences in cytokine titers between the control and experimental groups were determined with the Mann-Whitney U test.
